# Supplementary material for: Loss of dlx5a/dlx6a Locus Alters Non-Canonical Wnt Signaling and Meckel’s Cartilage Morphology
Source: Biomolecules. 2023 Sep 5;13(9):1347. doi: 10.3390/biom13091347 (PMC10526740; doi:10.3390/biom13091347)
Supplement: Supplementary file 1 [file biomolecules-13-01347-s001.zip › biomolecules-2570359-supplementary.pdf]

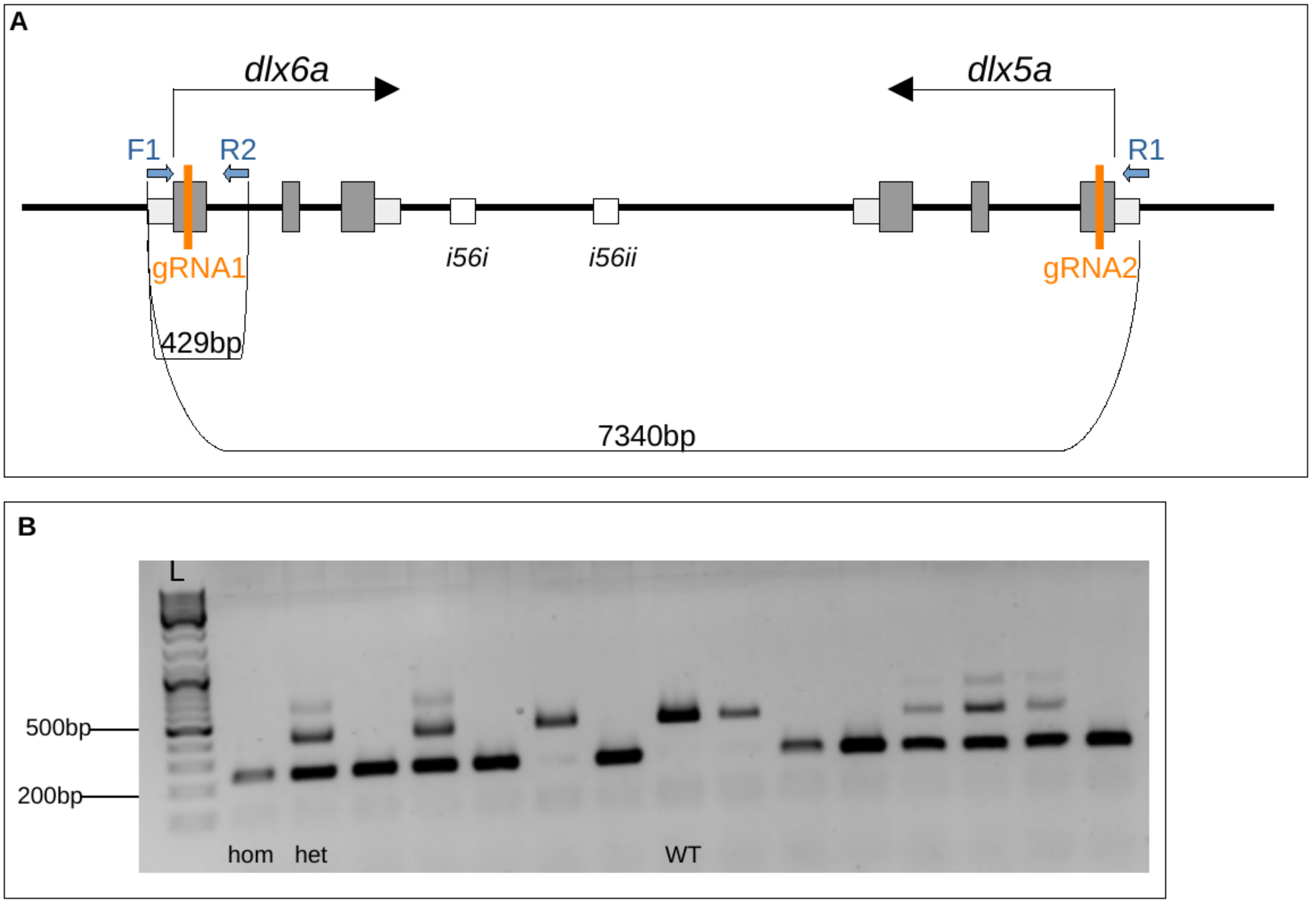

<u>Figure S1:</u> Creation of *dlx5i6* mutants using CRISPR-Cas9. (A) Schematic of mutagenesis strategy. Light gray boxes represent UTRs, dark gray boxes represent coding regions and white boxes represent cis-regulatory elements present in the intergenic region. Directionality of *dlx6a* and *dlx5a* are represented by the large arrows. Two gRNAs (orange boxes) were designed to target the first exons of *dlx6a* and *dlx5a*. These gRNAs were injected together with Cas9 protein into 1 cell stage embryos. To genotype, three primers were designed (blue arrow). F1/R1 will anneal to regions flanking the gRNAs and would not create a product unless cutting of both guides occurred. R2 anneals to intronic region after the first exon and will produce a band if the locus was left intact, or if gRNAs cut independently. (B) Representative genotyping gel of *dlx5i6+/-* incross demonstrating cutting of both guides produced a band at approximately 220 bp. L indicates ladder. Hom: homozygous, Het: heterozygous, WT: Wild-type

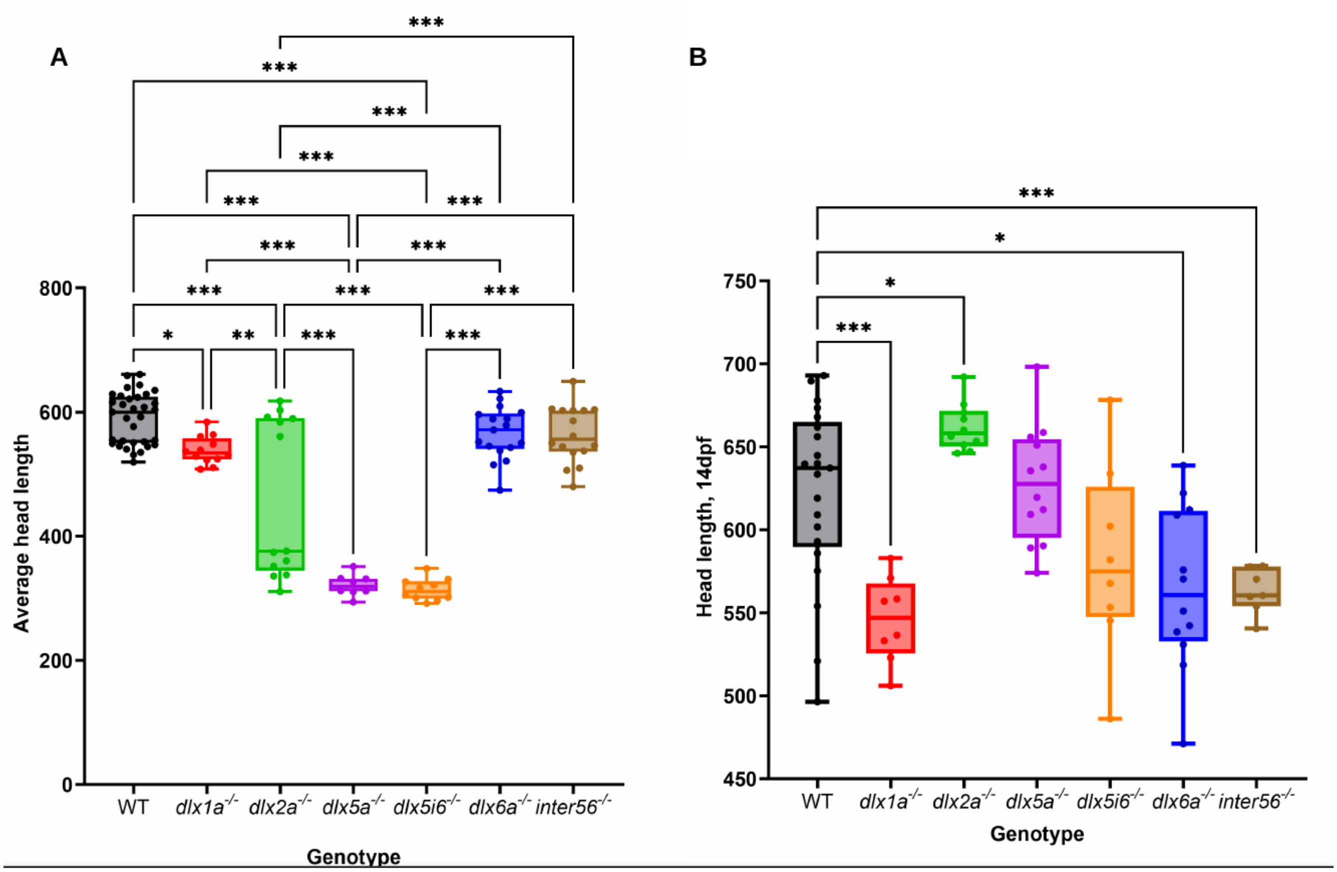

Figure S2: Average head lengths of *dlx*mutants at 5dpf and 14dpf. (A) Average head lengths at 5dpf for WT (n=33), *dlx1a*$^{-/-}$ (n=12), *dlx2a*$^{-/-}$ (n=14), *dlx5a*$^{-/-}$ (n=8), *dlx5i6*$^{-/-}$ (n=13), *dlx6a*$^{-/-}$ (n=17) and *inter56*$^{-/-}$ (n=16) larvae. (B) Average head lengths at 14dpf of *dlx* mutants where at least 8 animals were measured. Results are reported as mean ± SEM. One-way ANOVA with Tukey's multiple comparisons test was performed. *$p<0.05$; ** $p<0.001$; ***$p<0.001$

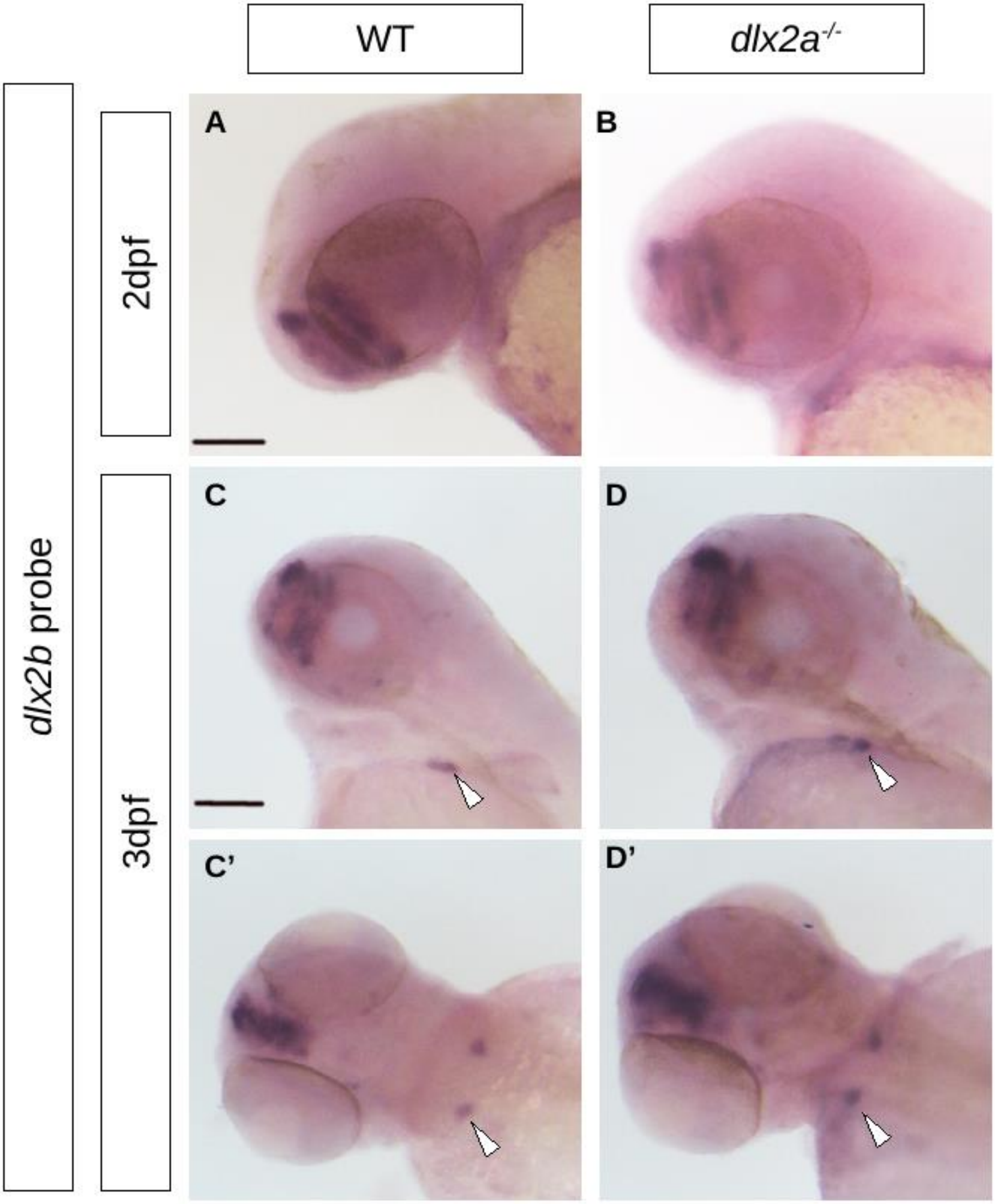

Figure S3: Expression of *dlx2b* in dlx2a mutant larvae at 2dpf and 3dpf. Expression is present in telencephalon, diencephalon and dental mesenchyme (arrowheads). (A, B) Expression in 2dpf *dlx2a* larvae. (C, D) Expression of dlx2b in 3dpf *dlx2a* mutant and WT sibling larvae in lateral and dorsal (C', D') positions. Scale = 100 µm.
